# *Helicobacter pylori* and the Role of Lipopolysaccharide Variation in Innate Immune Evasion

**DOI:** 10.3389/fimmu.2022.868225

**Published:** 2022-05-13

**Authors:** Daniel Sijmons, Andrew J. Guy, Anna K. Walduck, Paul A. Ramsland

**Affiliations:** ^1^ School of Science, RMIT University, Melbourne, VIC, Australia; ^2^ ZiP Diagnostics, Collingwood, VIC, Australia; ^3^ Department of Immunology, Monash University, Melbourne, VIC, Australia; ^4^ Department of Surgery, Austin Health, University of Melbourne, Heidelberg, VIC, Australia

**Keywords:** *H. pylori*, innate immunity, lipopolysaccharide, dendritic cells, Lewis system antigens, molecular mimicry, adhesion, inflammation

## Abstract

*Helicobacter pylori* is an important human pathogen that infects half the human population and can lead to significant clinical outcomes such as acute and chronic gastritis, duodenal ulcer, and gastric adenocarcinoma. To establish infection, *H. pylori* employs several mechanisms to overcome the innate and adaptive immune systems. *H. pylori* can modulate interleukin (IL) secretion and innate immune cell function by the action of several virulence factors such as VacA, CagA and the type IV secretion system. Additionally, *H. pylori* can modulate local dendritic cells (DC) negatively impacting the function of these cells, reducing the secretion of immune signaling molecules, and influencing the differentiation of CD4^+^ T helper cells causing a bias to Th1 type cells. Furthermore, the lipopolysaccharide (LPS) of *H. pylori* displays a high degree of phase variation and contains human blood group carbohydrate determinants such as the Lewis system antigens, which are proposed to be involved in molecular mimicry of the host. Lastly, the *H. pylori* group of outer membrane proteins such as BabA play an important role in attachment and interaction with host Lewis and other carbohydrate antigens. This review examines the various mechanisms that *H. pylori* utilises to evade the innate immune system as well as discussing how the structure of the *H. pylori* LPS plays a role in immune evasion.

## Introduction

*Helicobacter pylori* is a gram-negative, spiral shaped bacterium that infects the stomach of up to half the world’s population. The prevalence is dependent on country and can vary between 20% to 80% (1). *H. pylori* also has a high rate of clinically asymptomatic infection, with some studies reporting a prevalence as high as 67% in entirely asymptomatic populations (2, 3). However, chronic infection with *H. pylori* can result in severe clinical conditions, and colonisation induces chronic inflammation in all infected individuals (4). These conditions are thought to be caused by damage to the gastric epithelium and prolonged inflammation associated with chronic infection and immune action (3).

Antibiotics are the mainstay of treatment for confirmed infections, and quadruple antibiotic therapy is currently recommended (5). *H. pylori* strains are however increasingly found to be resistant and treatment failure is not uncommon (6). The outcome of *H. pylori* infection is strongly influenced by both the bacterial genotype as well as host polymorphisms and is driven by an array of virulence factors possessed by *H. pylori* (7). Approximately 15% of cases result in symptomatic gastric disease including acute gastritis, chronic gastritis, and peptic ulcer (8). Furthermore, 1-2% of infections result in malignant neoplastic diseases such as mucosal-associated lymphoid tissue (MALT) lymphoma and gastric adenocarcinoma (8). As a result of this *H. pylori* is classified as a type one carcinogen by the World Health Organisation (WHO) (9). The severity of *H. pylori* disease, and the increasing incidence of antibiotic resistance means there is an urgent need for new therapies and vaccines.

*H. pylori* employs several mechanisms to evade innate immunity allowing the bacterium to establish chronic infection. Firstly, *H. pylori* occupies a unique infective niche in the hostile conditions of the human stomach, using the secretion of the enzyme urease to survive the natural barrier of the stomach’s acidity, coupled with the motility *via* its unipolar flagella (10). The motility of *H. pylori* allows it to maintain position and reside in the gastric mucous gel layer where it triggers inflammation. The net effect of *H. pylori*-induced inflammation is damage to the epithelial barrier, which presumably releases nutrients to promote bacterial growth.

Despite chronic infection, inflammatory responses to *H. pylori* infection remain relatively controlled in most cases due to a series of mechanisms that manipulate the host response, promoting persistent infection. *H. pylori* is able to adhere to mucins such as MUC5AC (11), as well as interacting with Lewis system antigens expressed on gastric epithelial cells (12), this allows it to maintain its position in the mucus gel layer and crypt isthmus and prevent its removal by the stomachs churning movement (10). Secondly, *H. pylori* can interact with and manipulate interleukin (IL) secretion of local dendritic cells (DC), negatively regulating the functions of these cells and suppressing cytokine release (13), influencing the differentiation of CD4^+^ T helper cells towards Th1 type cells (14). Finally, the lipopolysaccharide (LPS) of *H. pylori* displays a high degree of phase variation and contains human blood group carbohydrate determinants such as the Lewis system antigens (15–17). This review examines the various mechanisms *H. pylori* utilises to evade the innate immune system as well as discussing how the structure of the *H. pylori* LPS plays a prominent role in immune evasion.

## Overview of Innate Immunity in the Context of Bacterial Infection

The innate immune system is the first line of defence against the many potential pathogens humans are exposed to daily (18). Epithelial surfaces and mucous membranes provide a physical barrier between internal organs and the external environment (19). The innate immune system also employs various antimicrobial peptides within the mucosal layer which have the ability to kill or inhibit microorganisms and viruses in the area (20). These initial defences are supported by more complex innate immune mechanisms. For example, pathogen-associated molecular patterns (PAMP) can be discerned from host molecular patterns by innate and adaptive immune cells allowing the triggering of a more extensive immune response (21). PAMP frequently include molecules such as glycans and glycoconjugates which may be expressed in structures like the LPS of a gram-negative bacterium (22). Detection of these structures are commonly associated with two classes of proteins known as toll-like receptors (TLR) and nucleotide-binding oligomerization domain-like receptors (NLR), as well as complement components circulating in the blood (23). The TLR expressed in humans are particularly well characterised, with TLR2, TLR4 and TLR5 commonly associated with the detection of lipopolysaccharide structures such as a bacterial LPS or flagella, whereas TLR3, TLR7, TLR8 and TLR9 are associated with the detection of foreign nucleic acids (24). Nod-Like Receptors recognise components of the bacterial cell wall, their activation triggers the release of pro-inflammatory cytokines and have synergistic effects with TLR in immune system signaling pathways (25).

Pathogen recognition triggers several supporting immune functions such as the activation of proinflammatory effector molecules. Inflammation is an important part of the immune response and inflammasomes are a system of innate immune receptors that activate the cystine protease caspase-1 and induce inflammation in response to the presence of infectious microbes (26). Additionally, inflammasomes contribute to the activation of proinflammatory cytokines IL-1β and IL-18, which induce an influx of innate immune cells into a potentially infected area, resulting in the secretion of further inflammatory cytokines, including IL-8, IL-6, IL-12, IL-17 and TNF-α, allowing for improved clearance of infection (26–28). Recognition of infectious bacteria often results in the engulfment of the bacterium by phagocytes including macrophages and monocytes. Engulfed bacteria are encased in an intracellular vesicle, the phagosome, where the phagocytic cells concentrate molecules which are deadly to bacterial cells, including antimicrobial substances, reactive oxygen species and reactive nitrogen intermediates. As well as immediate destruction, the engulfment, or phagocytosis of bacteria can also function as a form of antigen presentation for the activation of the adaptive immune system (29). A specialised type of phagocyte known as the dendritic cell (DC) plays a primary role in the activation of the adaptive immune system. Once a DC engulfs a bacterium, it processes the bacterial cell and presents its antigens in complex with major histocompatibility molecules (MHC) to lymphocytes to induce T cell and B cell maturation and differentiation, triggering the adaptive immune response (30).

## Immune Response Against *H. pylori*

Various innate immune mechanisms are activated by the colonisation of *H. pylori.* Toll-like receptors (TLR) 2, 4 and 5 can recognise PAMP of *H. pylori* (31), triggering immune pathways that activate NF-ĸB and IL-8 inducing a local proinflammatory response (32). *H. pylori* are extracellular and generally non-invasive, but secretion of urease and the VacA toxin trigger local inflammatory responses. The most researched virulence factor is the type IV secretion system (TIVSS) encoded by the *Cag* pathogenicity island carried by type I strains (33). After bacterial adherence the TIVSS delivers the cytotoxin CagA to the cytoplasm where it initiates intracellular signaling cascades that activate NF-κB (34), contributing to the local inflammatory response. The combined effect of this inflammation results in erosion of the epithelium, releasing nutrients for the bacteria to grow and colonise (35). Furthermore, intracellular pathogen recognising molecules such as NOD1 can bind *H. pylori* peptidoglycans that are introduced into epithelial cells *via* the TIVSS and trigger the release of other anti-microbial proteins in response, restricting bacterial growth and initiating additional immune action (36). While additional macrophages may be stimulated, *H. pylori* is able to neutralise macrophage nitric oxide production reducing the immune action of the cell (37). Once *H. pylori* has overcome the initial epithelial response, persistent infection drives an adaptive immune response triggered by dendritic cells. The local response consists of humoral and T cell (particularly Th1 and Th17) activation. Overall there is an influx of plasma cells, lymphocytes, neutrophils and other immune cells into the gastric mucosa during *H. pylori* infection, however this rarely results in complete clearance of the bacterium, and over time a significant population of regulatory T cells become established (38). This control of inflammation by *H. pylori* probably explains why many *H. pylori* infected individuals remain asymptomatic.

The stomach has relatively few polymeric immunoglobulin receptors (pIgR), the receptor responsible for IgA transportation, in comparison to the rest of the gastrointestinal tract (39). This is altered in chronic *H. pylori* infection with upregulation of the pIgR caused by raised γ-interferon levels associated with prolonged inflammation (40). Upregulation of pIgR does not result in a corresponding increase in local secretory IgA levels, with monomeric non-secretory IgA predominating in the stomach of those infected with *H. pylori* (41). In contrast, secretory IgA is commonly observed in response to intestinal commensals and pathogens, suggesting a different mode of action of pIgR in the stomach. Systemic *H. pylori* specific IgG is also produced in adults experiencing chronic infection (42).

### Role of *H. pylori* Virulence Factors in Induction of Innate Immune Responses and Immune Evasion

**Figure 1:**
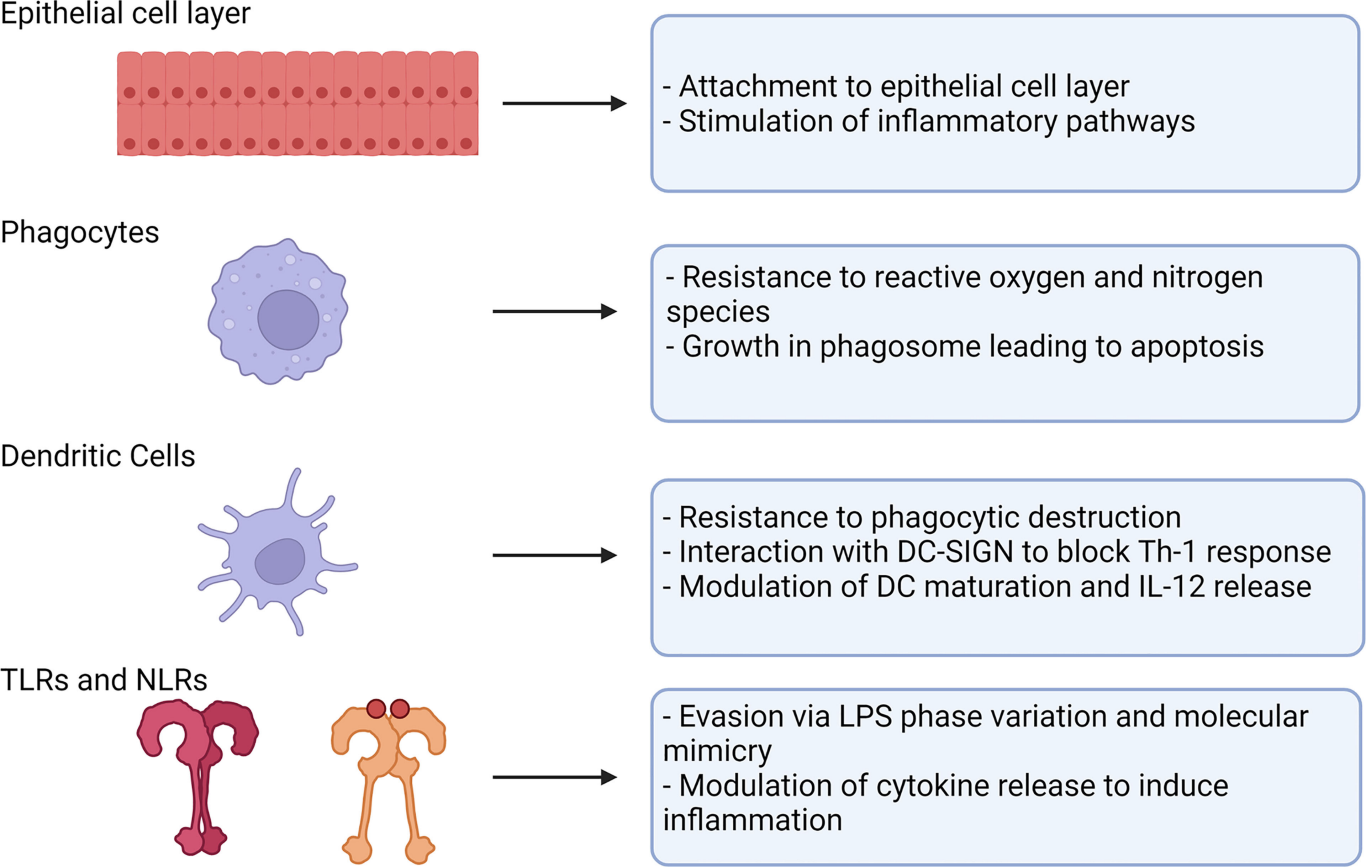
Strategies used by *H. pylori* to evade innate immune mechanisms. *H. pylori* uses a number of mechanisms to evade the innate immune system. Various components of the innate immune system present barriers to or respond to *H. pylori* infection (left). *H. pylori* is able to overcome these by mechanisms described (right). Created with BioRender.com.

The natural immune response against *H. pylori* does not effectively clear infection and a combination of immune evasion techniques and bacterial factors leads to a persistent infection (**Figure 1** and **Table 1**). Initial Type 1 inflammatory responses in most infected persons become biased over time towards Th2 and Treg that acts in a kind of damage control measure, which does not clear infection but reduces damage to the host (59–61).

**Table 1:** Effect of *H. pylori* virulence factors on the immune response.

Virulence factor	Immune evasion mechanisms	References
LPS	Evasion of pattern recognition receptors (TLR and NLR), inhibition of DC-SIGN.	(43)
BabA	Influences inflammasome activation. Prevention of removal by peristalsis and gastric shedding. Stimulation of inflammatory cytokines.	(44–47)
*cag* pathogenicity island and CagA	Prevention of phagocytosis and macrophage killing, regulation of DC cytokine production and inhibition of CD4^+^ T cell proliferation.	(13, 48, 49)
Flagellin	Evasion of TLR, evasion of peristalsis.	(50)
Urease	Neutralisation of acidity surrounding the bacterium, apoptosis of gastric epithelial cells.	(51, 52)
VacA	Prevention of phagocytosis and macrophage killing, interference with cytokines, alteration of antigen presentation, apoptosis of monocytes, inhibition of DC maturation, apoptosis of gastric epithelial cells.	(14, 49, 53–55)
*H. pylori* Neutrophil-activating protein (HP-NAP)	Neutrophil activation, increased production of inflammatory cytokines from neutrophils and monocytes and interference with Th2 responses.	(56, 57)
OipA	Induction of inflammatory cytokines.	(44, 45)
SabA	Nonopsonic activation of neutrophils, upregulation allowing for enhanced adhesion in inflamed gastric environments, down regulating enabling bacterial escape from strong host immune response.	(12, 58)

An important feature of *H. pylori* is its ability to overcome natural barriers preventing most microbes from colonising the human stomach (62, 63). Early studies concluded that *H. pylori* flagellin evades TLR5 recognition and recombinant *H. pylori* flagellin was considerably less stimulatory than *Salmonella* flagellin for example (64). Furthermore, a series of residues within the CD0 domain of *H. pylori* flagella protein FlaA has recently been found to enable evasion of TLR5 (**Figure 1**), these are speculated to be a result of point mutations not present in flagella proteins from other bacterium (65). However, a number of studies have now reported that *H. pylori* activates both TLR2 and 5 on epithelial cells, but not TLR4 (66). A recent report revealed that TLR5 activation is *via* interaction with the CagY protein which forms part of the TIVSS (67).

**Figure 2:**
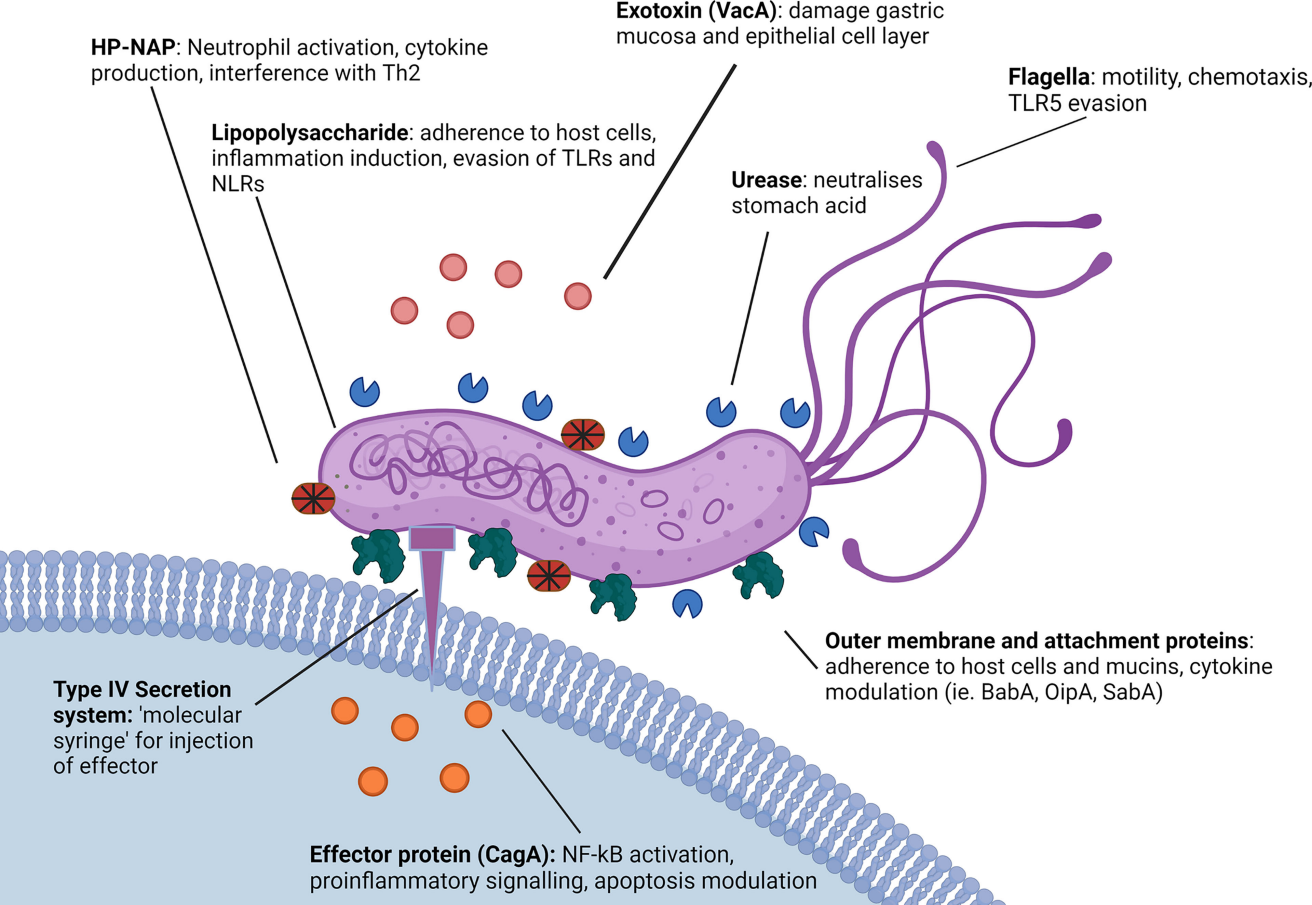
Role of *H. pylori* virulence factors in innate immune evasion. *H. pylori* utilises a number of virulence factors to enable immune evasion. *H. pylori* possesses a less immunogenic LPS in comparison to other gram-negative microbes and both the LPS and several *H. pylori* virulence proteins such as SabA and OipA demonstrate variable expression patterns affecting the immune response (68, 69). Additionally, *H. pylori* expresses proteins such as HP-NAP, VacA and CagA that actively modulate cytokine and inflammatory signaling, as well as DC and macrophage function (56, 70). *H. pylori* flagella protein FlaA also evades detection by TLR5 *via* a series of complex point mutations (65). Created with BioRender.com.

CagA is one of the most studied and most important virulence factors possessed by *H. pylori*. Contact and adherence of *H. pylori* to host gastric epithelial cells upregulates the expression of both CagA and VacA, as well as inducing the synthesis and assembly of the TIVSS (**Figure 2**) (71). Both CagA and the TIVSS are encoded by the *cag* PAI which is an approximately 40 kb chromosomal DNA region present in the most virulent strains of *H. pylori*. The TIVSS is a pilus-based “molecular syringe” structure that translocates CagA protein into host epithelial cells. The Type IV pilus binds epithelial cells *via* interaction with β1 integrin on the basolateral side (72). The bacteria gain access to the basolateral side through secretion of the HtrA enzyme which breaks down tight junctions (33).

Once in the cytoplasm, translocated CagA associates with Src and c-Abl family kinases which phosphorylate the CagA carboxyl-terminal EPIYA motif in the inner host cell membrane. Phosphorylated CagA then interferes with host cell signaling pathways, disrupting cell-cell junctions (73). In addition, CagA targets and causes the ubiquitination of the upstream kinase TAK1 (74). The downstream effects of CagA induce signaling cascades that activate NF-κB and downstream proinflammatory signaling (IL-8 secretion), cytoskeletal rearrangements, and changes to tight junctions (31, 33, 75) and inducing apoptosis (70, 76). Higher levels of CagA were shown to cause nuclear translocation of the AP-1 transcription factor and nuclear factor of activated T-cells (NFAT) (77). Furthermore, CagA is directly related to *H. pylori* carcinogenesis, enhancing DNA double strand breaks, and disabling homologous recombination-mediated DNA repair as well as stimulating Hippo signaling *via* the inhibition of PAR1b-mediated BRCA1 phosphorylation (78). Additionally, recent work has indicated that CagA initiates the oncogenic YAP pathway, further contributing to the association between CagA and gastric cancer risk (79, 80).

Infection with CagA positive *H. pylori* strains is one of the strongest risk factors for the development of gastric cancer (75). In addition, multiple *H. pylori* outer membrane proteins are associated with proinflammatory cytokine induction (**Figure 2**), these include OipA and BabA, which can induce IL-6, IL-8 and IL-11 (44, 45, 81). Like many bacteria, *H. pylori* have also been shown to release outer membrane vesicles (OMV) which contain various components, notably including portions of the *H. pylori* LPS as well as Hop family proteins including BabA and SabA along with other major virulence factors such as VacA and CagA (**Figure 2**) (74, 82). Interestingly, it appears that *H. pylori* is also able to regulate cytokine release from host epithelial cells *via* small noncoding RNAs that have been detected in OMV. These OMV have been shown to suppress IL-8 stimulation from AGS cells in cell culture, presumably by targeting host cell mRNAs, effectively reducing the overall immunostimulatory response to *H. pylori* LPS and outer membrane proteins (83). Consequently, OMV may play a role in immune modulation and disease progression. Regulation of cytokine release by *H. pylori* has also been investigated in relation to inflammasomes. *H. pylori* can modulate NLRP3, a regulator important for inflammasome activation, by inducing secretion of IL-10 in THP-1 monocytic leukemia cell lines, and through the action of miRNAs such as has-miR-223-3p (84). Furthermore, *H. pylori* infection was reported to suppress the secretion of mature IL-1β from human THP1 monocytes, in spite of upregulation of pro-IL-1β, potentially promoting bacterial persistence (85). Interestingly, cytokine expression has been seen to differ between children and adults, with Il-1β, IL17A, IL-23, IL2, IL-12p70 and IFN-γ upregulated in adults and downregulated in children, and IL-6, TGF-β1, IL-10, TNF-α and IL-1a downregulated in adults and upregulated in children. As a result of these differences, children display a Treg primary response as opposed to the Th1 and Th17 response seen in adults (86–88).

The virulence factor HP-NAP also plays a major role in inducing and modulating host inflammation, recruiting neutrophils to the site of infection and causing a large increase in the neutrophil secretion of reactive nitrogen species, as well as stimulating the release of IL-12 and IL-23 from both neutrophils and monocytes (89). *In vitro* studies using cloned CD4^+^ T cells from allergen induced T cell lines have shown HP-NAP acts as a Th2 agonist, redirecting Th2 responses into Th1 to drive further inflammation through cytotoxic Th1 responses characterised by increased TNF-α and interferon-γ (**Figure 2**) (56).

*H. pylori* is able to adhere to both the secreted gastric mucin MUC5AC (90) and gastric epithelial cells *via* several surface proteins, such as the blood group binding adhesin (BabA) and the sialic acid binding adhesin (SabA). These surface proteins interact with carbohydrate determinants such as the host Lewis system antigens, again providing a mechanism by which *H. pylori* prevents its removal by peristalsis as well as gastric shedding (**Figure 2**) (46).

*H. pylori* also expresses Lewis system antigens within the LPS, that is thought to represent a form of molecular mimicry which subverts the host immune response. Indeed, there is also evidence suggesting the expression of Lewis determinants is involved in *H. pylori* interacting with the DC calcium dependent (C-type) lectin DC-SIGN (**Table 1**) (91). Upon phagocytosis by an innate immune cell, *H. pylori* can also avoid destruction by interfering with phagosome maturation (**Figure 2**) (92). Another method of immune evasion used by *H. pylori* involves the interaction with DC (**Figure 1**). While *H. pylori* inhabits a unique niche in the stomach where few cells have access, there has been strong evidence of DC interaction, with DC maturation and function heavily affected in mouse models (93). Furthermore, there is evidence of *H. pylori* having the capability to infect DC in cell culture (48). *H. pylori* is capable of modulating IL-12 secretion by DC affecting the maturation and differentiation of CD4^+^ T cells and causing Th1 bias in mice (94). *In-vitro* studies have supported this, with *H. pylori* demonstrating the ability to modulate expression of IL-12 and TNF-α in cultured DC (95).

In addition to manipulating DC responses, other virulence factors including VacA (96) along with γ-glutamyltranspeptidase (GGT) (97) and CagA (98) have been reported to interfere with the T cell response to *H. pylori*, causing a characteristic hyposensitivity of CD4^+^ T cells and suppressing mucosal effector T cells (99). Notably, VacA has been found to have an influence on both DC cytokine expression and T cell differentiation, with VacA positive *H. pylori* strains showing the ability to supress IL-23 secretion by DC resulting in increased proliferation of Th17 cells. VacA plays additional roles in immune mediation including showing the ability to impair lysosomes and autophagosomes, a function with which VacA shares a synergistic effect with CagA, impairing the ability of both macrophages and gastric epithelial cells to remove internalised or invading bacteria. Specifically, *H. pylori* is able to inhibit the functions of lysosomal enzymes acid phosphatase, N-acetyl-β-d-glucosaminidase, and cathepsin D, prevent autolysosomal acidification, interfere with retrograde trafficking of mannose-6-phosphate receptors and inhibit autophagosome formation to promote intracellular survival of the bacterium (100).

It is likely *H. pylori* is highly resistant to destruction by phagocytes, although this has only been seen *in-vitro* (101). Even though *H. pylori* is frequently engulfed by phagocytes *in vitro*, it is proficient at neutralising and resisting the reactive oxygen and nitrate species released by monocytes and macrophages. This allows the bacteria to continue to replicate inside the phagosome, eventually inducing apoptosis of the host cell (49, 101). *H. pylori* possessing the *cag* pathogenicity island (*cag*PI) are even more resistant to phagocytosis than other strains (102), with isogenic mutants of *H. pylori* lacking *cag*PI far more readily taken up by phagocytes (49). The mechanism for this appears to be the ability of *H. pylori* to activate the reverse transsulfuration pathway to induce cystathionine γ-lyase (CTH) in host macrophages, promoting bacterial growth and enhancing bacterial survival in macrophages. *H. pylori* does this *via* the CagA-dependant induction of the PI3K/AKT1 pathway, the CagA-independent induction of the MTOR pathway and activation of SP1, increasing macrophage production of CTH (103) Furthermore, it has been shown that *H. pylori* is able to upregulate the metabolism of macrophage-associated polyamines, impairing M1 macrophage function (104).

### Antibody Activity and Humoral Immune Evasion by *H. pylori*

The mechanisms of humoral immune evasion by *H. pylori* are somewhat less clear. Most immunocompetent individuals who are infected with *H. pylori* develop a specific IgG and IgA response, in many cases high antibody titres to the pathogen, however, this often is not enough to clear infection (53, 105). Furthermore, it has been reported that the type of antibody produced correlates with the outcome of infection, with patients experiencing gastritis and duodenal ulcers having greater titres of IgG, and patients suffering from gastric cancers often having greater titres of IgA (106). Other studies have suggested that a weaker overall antibody response is linked to the development of gastric cancers, and patients who had a weak but still detectable antibody response to *H. pylori* had a higher rate of gastric cancer than those with a stronger antibody response (107–109).

## *H. pylori* LPS and Lewis Antigens

**Figure 3:**
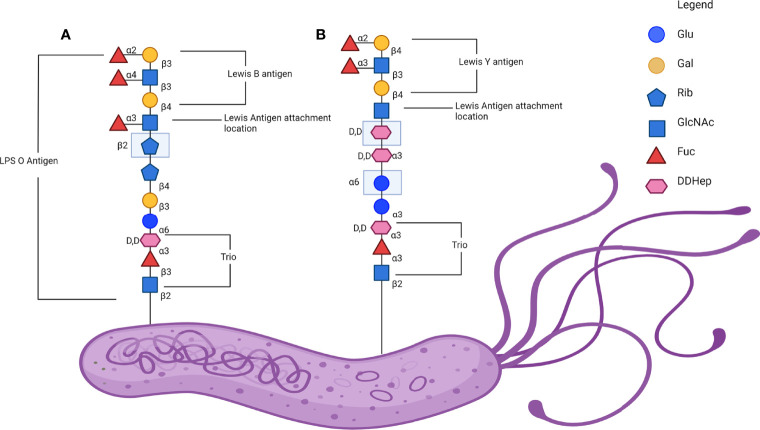
Lewis antigen expression on *H. pylori* lipopolysaccharide (LPS) O antigen. **(A)** A representative linear glycan chain of the *H. pylori* SS1 strain O antigen with the Le^b^ in comparison to **(B)** a representative linear glycan chain of *H. pylori* 26695 and G27 O antigen with the Le^y^ antigen attached. Adapted from the established structure in Li et al., 2018. Note that the exact structure and the Lewis determinate expressed is variable and dependant on isolate (115). Created with BioRender.com.

*H. pylori* both interacts with host Lewis system antigens and displays Lewis antigens in its LPS. The expression of Lewis antigens by *H. pylori* is associated with immune evasion and a reduced immunogenicity of its LPS when compared to other bacteria (110, 111). The reduced immunogenicity of *H. pylori* LPS is related to its structure, much like that of other gram-negative bacterium, the *H. pylori* LPS contains 3 domains: a hydrophobic lipid A domain on the outer bacterial membrane, a core oligosaccharide and a repetitive oligosaccharide termed the O chain (111). The O chain of the *H. pylori* LPS is the main location where Lewis system antigens are displayed by the organism (112). The *H. pylori* O antigen is typically composed of a Gal-GlcNAc backbone chain which is divided into two types based on its linkage (**Figure 3**). Type 1 chains are composed of Galβ1-3GlcNAc, which forms the core saccharide for Le^a^, sialyl-Le^a^ and Le^b^. Type 2 chains are composed of Galβ1-4GlcNAc or LacNAc, which forms the core saccharide for Le^x^, sialyl-Le^x^ and Le^y^ (63). In humans, Lewis system antigens like ABO blood group antigens are expressed in fluids and tissue including the gastric mucosa and endothelium. Le^a^ and Le^b^ are commonly expressed on various cell types from red blood cells to gastric epithelial cells, with Le^b^ in particular associated with various pathologies (113). In the human stomach, Le^a^ and Le^b^ are predominantly expressed on the surface and foveolar epithelia, whereas Le^x^ and Le^y^ are predominantly expressed in the mucus as well as the chief and parietal cells of the gastric glands. More specifically, non-secretory cells in the surface and foveolar epithelia express Le^a^ while Le^b^ and Le^y^ are expressed in secretory cells (114). Clinical isolates of *H. pylori* typically have a poly-LacNAc with several α-L-fucose residues forming internal Le^x^ determinants with terminal Le^x^ and Le^y^ determinants, while other strains have been described as displaying Le^a^, Le^b^ and sialyl-Le^x^ as well as group A, B and H-1 determinants (111). As many as 90% of clinical isolates of *H. pylori* contain Lewis antigens in the O antigen portion of the LPS. This expression has been found to be relatively stable after subculturing using methods such as immunoblot. However, it is possible for cultured *H. pylori* strains to lose Lewis antigen expression over time (68, 115).

### *H. pylori* LPS Structure, Phase Variation, and Diversity

As described above, the O antigen portion of the *H. pylori* LPS is associated with the expression of various Lewis antigens in addition to playing a role in the adhesion and immune evasion of the bacterium (116). The O antigen chain is comprised of a glucan group (saccharide composed of glucose), a D-glycerol-D-manno-heptan (DD-heptan) group and a highly conserved trisaccharide (trio) (115). Variability in the O antigen is derived from phase variation, whereby *H. pylori* uses an on/off system to regulate its biosynthetic genes, including the fucosyltransferase genes FutA, FutB, and FutC (117), allowing the bacterium to adjust the carbohydrate expression of the O antigen with the changing environment of the stomach and gastric mucosa (16). Additionally, phase variation of the *H. pylori* LPS allows for a greater range of phenotypes and gives *H. pylori* the ability to modify the expression of Lewis system antigens in its LPS. Additionally, it has been indicated that the *H. pylori* LPS induces lower biological responses when compared to other gram-negative organisms such as *E. coli* and *Salmonella* spp., with *in vivo* mouse and rabbit studies showing 500 to 1000 fold lower mitogenic and pyrogenic responses (118).

The genes encoding the glycotransferases responsible for the construction of this LPS vary between different regional strains, with the common European strain of *H. pylori* G27 possessing the trisaccharide fucosyltransferase (HP0102), heptan transferase (HP1283) and GlcNAc transferase (HP1578), the last of which is responsible for initiating synthesis of Lewis system antigens onto a heptan motif (111). In addition to this, a comparison between the European model strain G27 and East Asian strains found that the East Asian strains lacked the genes encoding for heptan transferase and GlcNAc transferase (119). Furthermore, East Asian strains instead express additional copies of other genes, HP1105 and JHP0562, which may act as GlcNAc transferases as well as Gal transferase in place of HP1578 (119). This was identified as an area of interest due to the higher rates of gastric cancer in East Asia and the potential to further characterise the role of the *H. pylori* LPS heptan in pathogenesis (119). **Figure 3** illustrates an abbreviated version of the established structure of the O antigen structure from the mouse-adapted *H. pylori* SS1 strain including the trio, attachment site and the Lewis antigenic determinants.

### *H. pylori* LPS Structural Diversity in Infection and Immune Evasion

The *H. pylori* LPS is generally considered less immunogenic in comparison to enterobacterial LPS, with early studies showing significant reductions in pyrogenicity, mitogenicity and toxicity, as well as a reduced cytokine and chemokine response (118, 120, 121). Previous studies have shown that synthesised partial *H. pylori* LPS structures, specifically lipid A compounds and Kdo-lipid A compounds, can modulate cytokine production by host cells. All lipid A and Kdo-lipid A *H. pylori* LPS structures synthesised either failed to induce, or induced very low levels of IL-1β, IL-6 and IL-8, and conversely stimulated high levels of cytokines IL-12 and IL-18 in heparinised human peripheral whole blood (122, 123). IL-12 induction by *H. pylori* is generally linked to MyD88 expression in macrophages (124). Furthermore, *H. pylori* modifies the lipid A portion of the LPS by *via* dephosphorylation of the 1- and 4-’positions of the lipid A backbone. Mutations to the lpxE/F machinery required for this modification have shown an increased susceptibility of *H. pylori* to the antimicrobial peptide polymyxin B, as well as reducing the ability of *H. pylori* to colonise mice (125). Knockout studies on *H. pylori* genes *HP0044* and *HP1275* demonstrated the production of a truncated *H. pylori* LPS missing fucose residues found in both the trio and Lewis antigen portions of the *H. pylori* LPS O antigen, resulting in the loss of a significant portion of the *H. pylori* LPS. This in turn affected the growth of the bacterium, increased its susceptibility to both the detergent SDS, promoted bacterial autoaggregation, increased surface hydrophobicity and affected bacterial virulence and OMV protein sorting (126). Notably, phase variation of *H. pylori* fucosyltransferases, and consequently the glycosylation pattern of the LPS has previously been identified to occur in high frequencies, with *in-vitro* studies demonstrating the expression of Le^x^ can vary at a frequency of 0.2-0.5% resulting in differing LPS variants in the same population. Variation of Lewis antigen expression, particularly between Le^x^ and Le^y^ has been suggested to be influenced by relative pH in liquid medium. The function for this has not been fully identified, but is hypothesised to aid in bacterial persistence (127). On a molecular level the variation in *H. pylori* fucosylation has previously been attributed to slipped-strand mispairing as a result of differing numbers of polyC repeats in fucosyltransferase genes *futA*, *futB* and *futC.* However, a recent study has described a role for small RNAs (sRNA) in the regulation of the *H. pylori* LPS biosynthesis. The sRNA RepG modulate *HP0102* and *TlpB*, providing post-transcriptional regulation to LPS biosynthesis potentially indicating further means by which *H. pylori* is able to adapt to host immune response (128).

## Role of *H. pylori* Adhesins in Immune Evasion

In addition to *H. pylori* commonly expressing Lewis determinants in its LPS, *H. pylori* also binds to host Lewis determinants, assisting in the attachment of the bacterium to the gastric epithelium (129, 130). Binding is facilitated by a group of outer membrane proteins, including BabA and SabA.

### BabA

*H. pylori* binds to the various Lewis system antigens expressed on the surface of gastric epithelial cells, including Le^b^ and sialyl-Le^x^ (131). Chronic inflammation also leads to upregulation of Le^x^ and Le^y^ in the host, further enhancing bacterial colonisation (131). Furthermore, *H. pylori* has a range of adhesive proteins that have been identified and examined. The first identified was BabA, a protein that commonly binds the Le^b^ determinant as well as mucin proteins and the H1 blood group antigen (132). Specifically, BabA binds the lacto series of glycans containing a terminal fucose molecule with an α1,2 linkage attached to a Galβ1-3GlcNAc core (133). This includes H1 blood group antigen as well as other H neoglycoconjugates and Le^b^. However, BabA binds to Le^b^ with as much as a two-fold increase in affinity in comparison to H neoglycoconjugates (134). The BabA encoding gene(s) can be found in three separate loci on the *H. pylori* genome with the binding pattern of the protein differing based on the specific loci expressed (135). For example, strains containing locus A BabA (BabA2) bind the classically associated Le^b^ whereas strains with locus B BabA (BabA1) do not bind to Le^b^ (136). Furthermore, some strains may contain more than one BabA encoding gene allowing expression of proteins with varying binding characteristics (137). BabA in general is a highly polymorphic protein with variable expression, which may be lost during laboratory cultivation. Additionally about one quarter of strains isolated from chronically infected hosts tend to lose BabA expression (69, 138). This suggests that BabA is a specific adhesin that is advantageous in the colonisation of a human host that is unnecessary on solid media (137). There has been evidence of numerous binding molecules for BabA in addition to the Le^b^ molecules expressed by gastric epithelia. Several salivary proteins have also been identified to interact with BabA, such as the mucin MUC5B, the agglutinin glycoprotein-340 and the proline-rich glycoprotein containing the Fucα1-2Galβ motif. In addition to this, fucose-containing oligosaccharides present in secretory IgA also play a role in binding BabA, however this is not universal to all secretory IgA (132). It has been observed that BabA presence in a *H. pylori* strain is associated with expression of VacA, CagA and OipA among other common but variable virulence factors, particularly in the outcome of gastric metaplasia. An *in-vitro* study found that Le^b^-positive AGS cells expressed increased levels of mRNA for the cytokines CCL5 and IL-8 as well as precancer related factors CDX2 and MUC2 in the presence of wild type *H. pylori*, however this was not the case for *H. pylori* mutants with either BabA genes or TIVSS deleted (139).

### SabA

SabA is an additional adhesion molecule associated with the binding of Lewis system determinants by *H. pylori.* More specifically, SabA binds sialylated molecules such as sialyl-Le^x^ and some gangliosides characterised by Neu5Acα3-neolactohexaosylceramide and Neu5Acα3-neolactooctaosylceramide molecules (58). The gastric inflammation caused by *H. pylori* infection is essential for changes in glycosylation patterns within the gastric mucosa, promoting the expression of sialyl-Le^x^ as well as sialyl-Le^a^, which in turn increases the adhesive properties of SabA (140). Similar to BabA, the expression of SabA is highly variable and is often subject to phase variation. It has been suggested that *H. pylori* may have multiple means of modulating the expression of SabA, with one such method being an acid-responsive ArsRS two-component signal transduction system, and an alternative involving the slipped-strand mispairing of SabA alleles during chromosomal replication in various *H. pylori* subpopulations (141). Additionally, SabA is commonly subject to homologous recombination and gene conversion due to changing environmental pressures (142). Deactivation of the SabA encoding gene may further assist in bacterial escape as cytotoxic activity by neutrophils is in part triggered by SabA binding to gangliosides. Neutrophil action has been shown to be defective against SabA deleted *H. pylori* cells (58).

### OipA

OipA is also a member of the Hop family of outer membrane proteins possessed by *H. pylori.* There is currently no available crystal structure for OipA, limiting detailed studies of its binding (143). OipA is known to be closely associated with the *cag* PAI and CagA, with the *oipA* locus approximately 100 kb from the *cag* PAI. OipA is regulated by slipped-strand mispairing and displays on/off states which are closely associated with the expression of CagA. Alleles of *oipA* have been demonstrated in up to 96% of *cag* PAI positive *H. pylori* strains, solidifying the relationship of the two virulence factors (144). With the exception of CagA, OipA is typically independent of other *H. pylori* virulence factors. OipA is associated with the induction of IL-8 from host epithelial cells, inhibition of apoptosis and enhanced adhesion to gastric cells *in vitro* (144). Induction of IL-8 and its subsequent role in inducing gastritis has been associated with a synergistic effect from *H. pylori* strains possessing both the *cag* PAI and a functional *oipA* gene (145). In contrast *oipA* “off” strains can down-regulate anti-apoptotic processes in AGS cells, more regularly inducing apoptosis on infection than *oipA* “on” strains *in-vitro* (146).

## Conclusions

*H. pylori* is a very successful global pathogen and has adopted a range of adaptions to evade the innate immune system. These mechanisms include both active systems, such as the neutralisation of reactive oxygen and nitrogen species released by macrophages, the modulation of cytokine secretion and the maturation of dendritic cells, and more passive systems such as the variability and the uniquely low immunogenicity of its LPS (15, 53). In addition, the expression of Lewis antigens in the LPS of *H. pylori* gives the bacterium the ability to mimic host antigens and thereby hide from the immune system (147). Notably, several of the immune evasion mechanisms *H. pylori* employs are yet to be fully explained. For example, knowledge of *H. pylori* activation and modulation of innate immunity outside of interactions with dendritic cells and DC-SIGN is surprisingly limited. It is also known that outer membrane proteins such as BabA, SabA and OipA play a role in the induction of inflammatory cytokines, although the molecular mechanism is still unclear (44, 144). Further study into ways to circumvent the methods *H. pylori* uses for immune evasion could allow for improved treatment and vaccination options. Additionally, blocking the activity of *H. pylori* adhesins to prevent attachment in the gastric mucosal gel layer and to epithelial cells has potential for new treatment options in an environment of increased antibiotic resistance.

## Author Contributions

DS, prepared figures and wrote the manuscript; AG, critically reviewed and commented on the manuscript; AW, critically reviewed and commented on the manuscript; PR, critically reviewed and commented on the manuscript. All authors conceived and discussed the topic of the review. All authors read and approved the final manuscript.

## Funding

DS is supported by a Research Training Program Stipend Scholarship from the Australian Government, Department of Education and Training.

## Conflict of Interest

Author AG was employed by company ZiP Diagnostics.

The remaining authors declare that the research was conducted in the absence of any commercial or financial relationships that could be construed as a potential conflict of interest.

## Publisher’s Note

All claims expressed in this article are solely those of the authors and do not necessarily represent those of their affiliated organizations, or those of the publisher, the editors and the reviewers. Any product that may be evaluated in this article, or claim that may be made by its manufacturer, is not guaranteed or endorsed by the publisher.
